# Gliotoxin, an Immunosuppressive Fungal Metabolite, Primes Plant Immunity: Evidence from *Trichoderma virens*-Tomato Interaction

**DOI:** 10.1128/mbio.00389-22

**Published:** 2022-07-18

**Authors:** Rinat Zaid, Roni Koren, Efrat Kligun, Rupali Gupta, Meirav Leibman-Markus, Prasun K. Mukherjee, Charles M. Kenerley, Maya Bar, Benjamin A. Horwitz

**Affiliations:** a Faculty of Biology, Technion – Israel Institute of Technology, Haifa 3200000, Israel; b Nuclear Agriculture and Biotechnology Division, Bhabha Atomic Research Centre, Mumbai, India; c Department of Plant Pathology and Microbiology, Texas A&M University, College Station, Texas, USA; d Department of Plant Pathology and Weed Research, ARO, Volcani Institute, Rishon LeZion, Israel; Tel Aviv University; Tel Aviv University

**Keywords:** Trichoderma, gliotoxin, immunity, plant symbiont, root, tomato

## Abstract

Beneficial interaction of members of the fungal genus *Trichoderma* with plant roots primes the plant immune system, promoting systemic resistance to pathogen infection. Some strains of *Trichoderma virens* produce gliotoxin, a fungal epidithiodioxopiperazine (ETP)-type secondary metabolite that is toxic to animal cells. It induces apoptosis, prevents NF-κB activation via the inhibition of the proteasome, and has immunosuppressive properties. Gliotoxin is known to be involved in the antagonism of rhizosphere microorganisms. To investigate whether this metabolite has a role in the interaction of *Trichoderma* with plant roots, we compared gliotoxin-producing and nonproducing *T. virens* strains. Both colonize the root surface and outer layers, but they have differential effects on root growth and architecture. The responses of tomato plants to a pathogen challenge were followed at several levels: lesion development, levels of ethylene, and reactive oxygen species. The transcriptomic signature of the shoot tissue in response to root interaction with producing and nonproducing *T. virens* strains was monitored. Gliotoxin producers provided stronger protection against foliar pathogens, compared to nonproducing strains. This was reflected in the transcriptomic signature, which showed the induction of defense-related genes. Two markers of plant defense response, PR1 and Pti-5, were differentially induced in response to pure gliotoxin. Gliotoxin thus acts as a microbial signal, which the plant immune system recognizes, directly or indirectly, to promote a defense response.

## INTRODUCTION

Gliotoxin, a non-ribosomal peptide metabolite of fungal origin, was the second “antibiotic” to be discovered after penicillin, and the producing strains of Trichoderma virens were extensively evaluated as plant disease biocontrol agents, as gliotoxin is highly toxic to some plant pathogens, including Rhizoctonia solani and *Pythium* spp. The discovery of gliotoxin in an opportunistic human pathogen and the establishment of its role in virulence, however, subsequently led to this strong antimicrobial metabolite being designated as a mycotoxin (1, 2). In our earlier study, using gene deletion, the role of gliotoxin in direct antagonism against plant pathogens was established (3). In animal models, damage to cells is well-documented, as are the induction of apoptosis and interferences with NF-κB signaling and proteasome function. The mechanism is, at least in part, oxidative stress catalyzed by gliotoxin, which is a redox-active molecule (1). Immunosuppression is apparently the result of this multiple damage, which interferes with neutrophil activity. Invasive aspergillosis leads to gliotoxin levels that affect human neutrophils. Malcolm et al. (4) noted inhibition of phagocytosis, actin reorganization, and cell shrinkage, as well as loss of filipodia, all of which would favor the pathogen.

Like many other plant-associated *Trichoderma* spp., T. virens is a symbiotic fungus that promotes growth and immunity. The association of plant roots with members of the fungal genus *Trichoderma* systemically primes the plant immune system against infection (5–9). Priming, which usually brings to mind bacteria and fungi, is effective even against nematodes (10) and insect pests. Tomato plants primed by T. atroviride, for example, displayed stronger direct defense against an aphid pest, as well as produced volatiles that attracted a parasitoid wasp (11). In plant immunity, there is often a tradeoff between growth and defense responses (12, 13), though some strains of *Trichoderma* can simultaneously prime plant immunity and promote growth (14, 15). Plant roots apparently detect the fungus, at first, as an invader. In this stage, *Trichoderma* must evade plant defense (16) to colonize the root epidermis and outer cortex layers. In parallel, it must trigger some immune response to potentiate systemic resistance. One type of effector for systemic resistance is represented by Sm1/Epl1 (17, 18). To select (or even design) the best strains for agricultural biocontrol, we need to better differentiate between mutualists and pathogens in the rhizosphere (19). To the extent that mutualists trigger some of the molecular machinery that the plant uses to defend itself against pathogens, they can prime systemic resistance through plant defense (6, 20, 21). Plant immunity consists of two main components: pattern-triggered immunity (PTI) and effector-triggered immunity (ETI) (22). The PTI/ETI dichotomy is not as sharp as first thought, as the two overlap, even at the receptor level (23, 24). *Trichoderma*-root interactions depend on the species and strain of both the plant and the fungal partners (25, 26). The widespread ability of *Trichoderma* spp. to interact with different plant species, including important crops, suggests that a single effector type is unlikely to underlie the broad host range. On the contrary, different studies point to a multitude of secreted molecules. These include enzymes, whose activities are, in some cases, dispensable for their actions as effectors (27–29), as well as small secreted cysteine-rich proteins (SSCPs), such as the ceratoplatanin family member, Sm1/EPL1 (9, 17, 18).

Although it is often assumed that the pattern receptor ligands of the PTI and the effectors of the ETI are proteins, small molecules (metabolites) also contribute to the fungal-plant dialog. Indeed, extensive changes in the plant hormone balance occur, resulting from active plant growth modulators produced by both the fungal and the plant partners (8). The extensive array of secondary (specialized) metabolites produced by fungi are also relevant. Genetic evidence predicted that a secondary metabolite produced by rice blast ACE1 (Avirulence Conferring Enzyme, a hybrid PKS-NRPS) is a virulence factor recognized by the host in a gene-for-gene interaction (30, 31). Though not pathogens, *Trichoderma* spp. produce secondary metabolites that, likewise, participate in interactions with the plant host (32–35). The metabolic patterns of maize roots colonized by *T. virens* differ extensively from those of non-colonized roots, and the metabolome of colonized roots depended on two secondary metabolism-related genes (36).

Trichoderma virens produces gliotoxin, the product of a biosynthetic cluster defined by its non-ribosomal peptide synthetase (NRPS) gene, *GliP*. This cluster is present in “Q” strains which include the sequenced reference strain, Gv29-8 (37). A transcriptomic study highlighted gliotoxin synthesis in the mycoparasitism of T. virens, while T. atroviride seems to rely more on cell wall degrading enzymes (38). Vargas et al. (3) generated mutants at the *GliP* locus. Lacking gliotoxin, these mutants, among other phenotypes, lost the ability to attack some soilborne pathogens but not others. The loss of this direct antagonism was reflected in the loss of protection of cotton seedlings against these same pathogens. Since there is not much information on the role of gliotoxin in direct plant interactions, especially about its effects on plant immunity, we investigated whether gliotoxin participates in the indirect protection of a plant host in which interaction with *Trichoderma* systemically primes plant immunity.

While optimizing the *Trichoderma*-plant plate assay with Arabidopsis seedlings (39) for Q strain Gv29-8, we noticed that this strain not only failed to promote growth but also overgrew and killed the seedlings, though this did not occur with soil-grown plants (40). This strain was previously reported to inhibit the growth of tomato seedlings, with a decrease of about 20% in root and stem length relative to controls (41). The authors proposed that one contributing factor is the phytotoxicity of gliotoxin (1, 41, 42). T. virens Gv29-8 also suppressed the growth of maize roots (36). As Arabidopsis seedlings are exceedingly small and sensitive, we tested the role of gliotoxin production on the induction of systemic resistance and plant growth, using tomato as the host. Growth promotion of tomato depends on the *Trichoderma* strain/species (25, 26). Mutants were earlier constructed, carrying a deletion in the signature NRPS gene of the gliotoxin biosynthesis cluster, *GliP*. Gliotoxin was undetectable in the mutants, which were defective in their direct antagonism against some oomycete and fungal hosts, and were ineffective in control of cotton seedlings against the soil-borne pathogen, Pythium ultimum (3). Here, we compared the responses of tomato seedlings to three T. virens strains: the wild type Gv29-8, a *ΔgliP* mutant, and a gliotoxin nonproducer. We found a central role of gliotoxin in triggering the plant immune response relevant to priming against infection by foliar pathogens. We also investigated the importance of gliotoxin in the reprogramming of the leaf transcriptome by the interaction of tomato roots with the fungal partner.

## RESULTS

### Gliotoxin production by *Trichoderma* inhibits growth of tomato seedlings.

**FIG 1:**
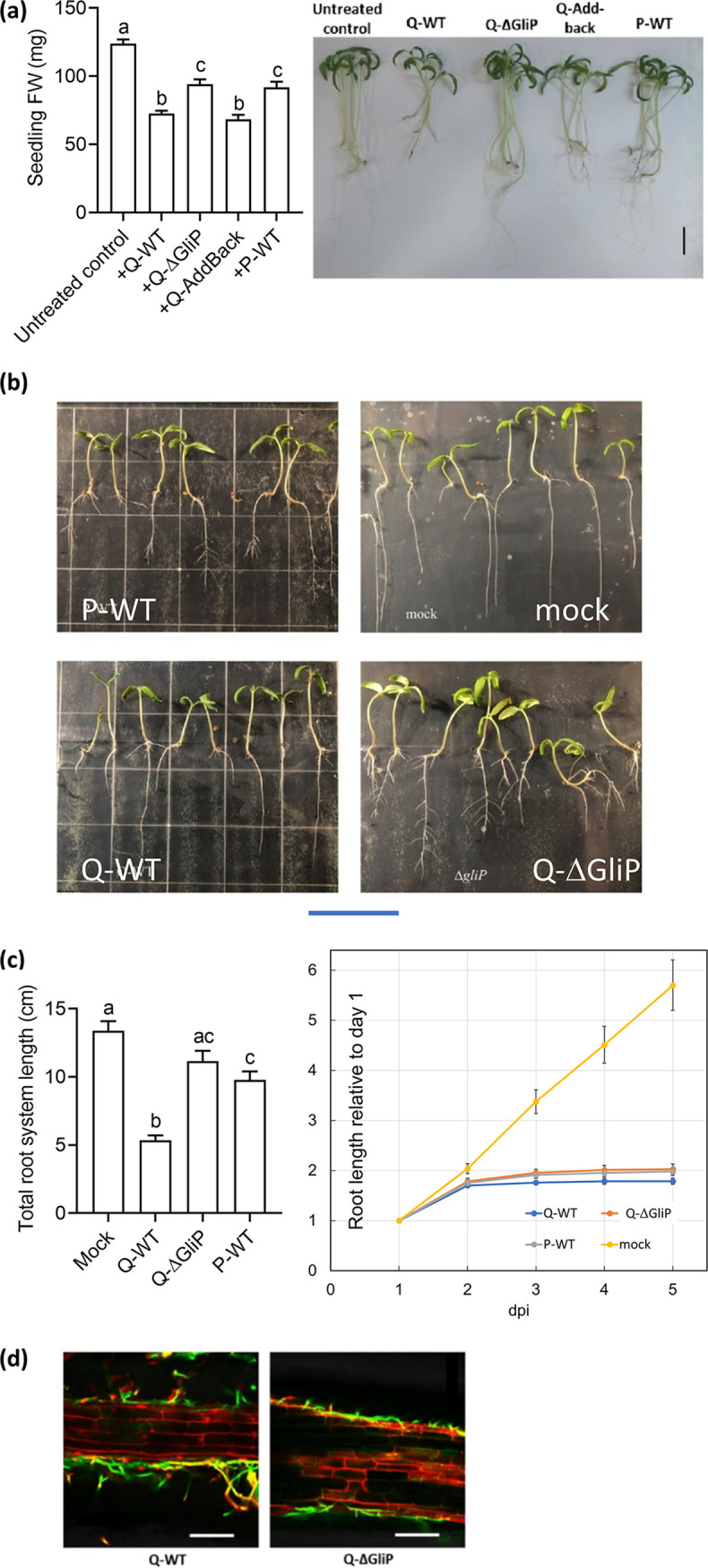
Biomass and root development of tomato seedlings interacting with gliotoxin-producing and nonproducer *T. virens* strains. 12-day-old seedlings (5 per box) were grown in ½ MS-agar magenta boxes or large plates. The seedlings were treated with spore suspensions of the appropriate fungal strain dripped on the agar surface. The following strains were tested: *T. virens* Gv29-8 (Q-WT), gliotoxin-deficient mutants in the same genetic background (Q-ΔGliP), complemented control (Q-Addback), and *T. virens* IMI 304061 (P-WT), or with sterile water as a control. (a) Seedling biomass at 9 days post-inoculation (dpi). Error bars indicate SEM. Different letters indicate significant differences between strains and treatments for 3 experiments. Right, representative image of seedlings from the magenta box assay, 2 weeks after *Trichoderma* inoculation. Scale bar = 2 cm. (b) Root system development on large, vertically-oriented plates in interaction with gliotoxin-producing and nonproducing strains. Scale bar = 7 cm. (c) Suppression of primary root growth by *Trichoderma* strains. Left, total root system length at 5 dpi. Bars indicate means for 52 to 65 seedlings per treatment from a total of 3 independent experiments. Different letters indicate significant differences. Right, primary root length relative to the length at 1 dpi. Error bars indicate SEM. (d) Representative confocal images of tomato root colonization by *T. virens* and its gliotoxin-lacking mutant (Q-*ΔgliP*), 72 hpi. Green channel, Alexa-fluor WGA; red channel, propidium iodide. Scale bar = 100 μm.

Interaction with the T. virens Q strain Gv29-8 caused a decrease of about 2-fold in the total biomass accumulated at 2 weeks. The shoots of tomato seedlings treated with the Q-WT in sterile culture appeared normal, although having decreased biomass (Fig. 1a). The root system, however, was damaged after long growth times, and the removal of the seedlings from the agar substrate often broke the primary tap root. These negative effects on growth are primarily the result of gliotoxin production, as they are lacking in the mutant and the P WT strain, neither of which produce gliotoxin. The “addback” strain, complemented by the Aspergillus fumigatus *GliP* ortholog (4), showed nearly complete rescue of the gliotoxin-related growth-suppression phenotype. To better quantitate the effects on the root system, seedlings were grown on large, vertically-oriented plastic culture plates. In this configuration, both the Q and P WT strains suppressed elongation of the tap root and of basal and shoot-borne roots. P and Q *ΔgliP*, however, strongly promoted lateral root formation such that, overall, the total length of the root system was similar to non-inoculated control seedlings (Fig. 1b–d). Q *ΔgliP* and the P WT caused a modest suppression of total root system length. This effect was significant for the P strain. As illustrated in Fig. 1b, root system architecture differed strikingly between the controls and plants inoculated with the gliotoxin producer, Q-WT, and the nonproducers, Q-*ΔgliP* or P-WT. The suppression of primary root elongation and the promotion of lateral root formation have been reported previously: *Trichoderma* produces volatiles and auxins that modulate plant growth, and the effect of a given strain varies, depending on the host and environmental conditions (36, 39, 43, 44). Both WT and *ΔgliP* colonized roots. Some propidium iodide staining of root epidermal cell nuclei, indicating cell death, was often observed in interaction with all *Trichoderma* strains (Fig. 1d).

### Gliotoxin producing capability is essential for induced defense against *B. cinerea* and *X. euvesicatoria*.

**FIG 2:**
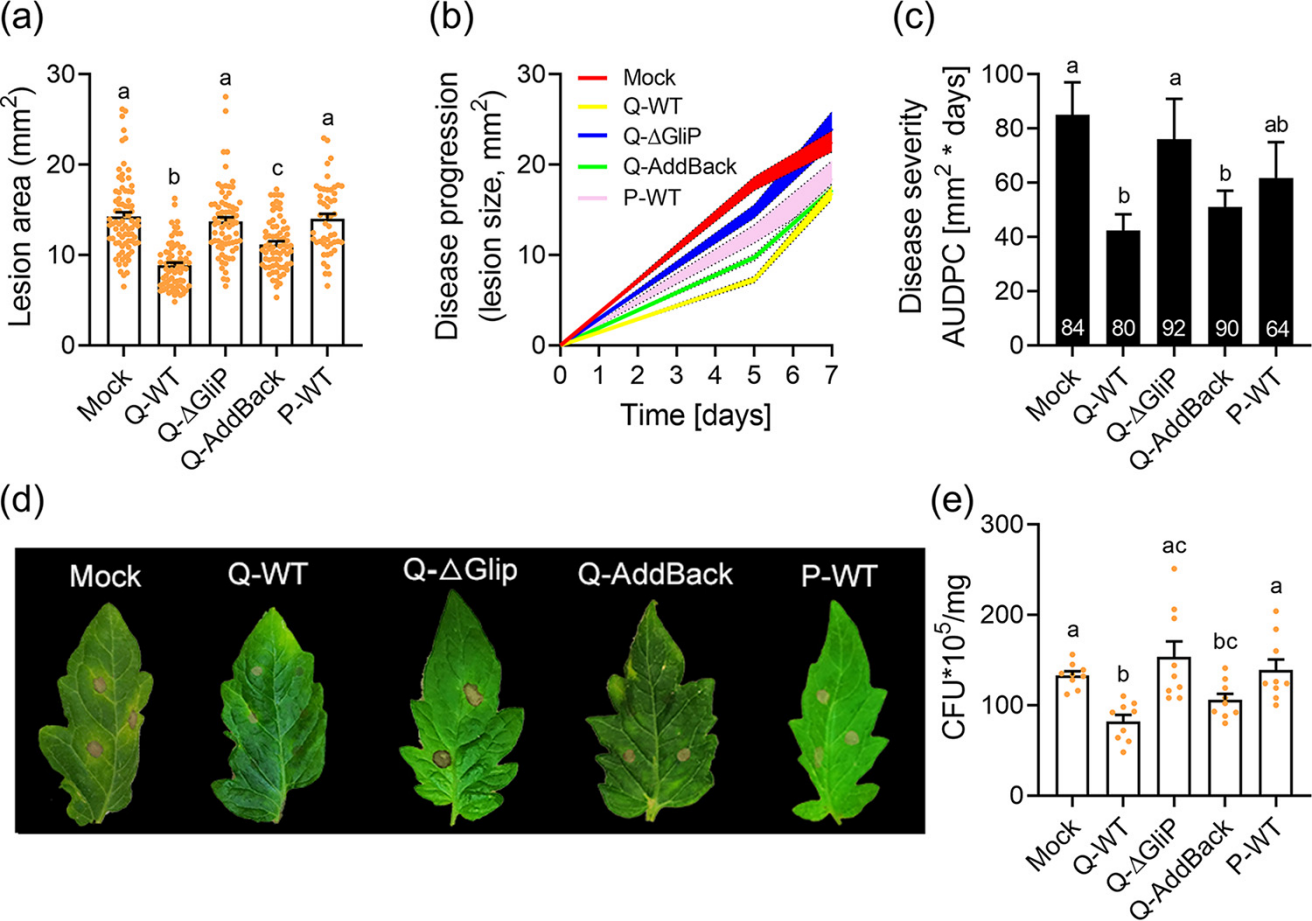
Analysis of disease protection by ISR, conferred by root treatment with different *T. virens* genotypes. (a-d) Botrytis cinerea: 5- to 7-week-old plants were soil drenched with a spore solution of the indicated *T. virens* genotypes (10^7^ spores mL^−1^) twice, then inoculated with 10 μL droplets of a *B. cinerea* spore solution (10^6^ spores mL^−1^) 2 h after the second drench. Plants treated with water were used as mock. (a) Lesion area was measured 5 days after *B. cinerea* inoculation using ImageJ. Graph represents the results of 3 independent experiments ± SE, N ≥ 64 for each treatment. Letters indicate significance in a one-way ANOVA with a Dunnett’s post hoc test, *P* < 0.0001. (b) Lesion area was measured using ImageJ every 2 days for a week; average lesion size ± SE is plotted against time. (c) Total area under the disease progression curve (AUDPC) in the different treatments. Experiment was conducted 3 times with similar results. Graph represents the results of 3 independent experiments ± SE, N numbers for each treatment are indicated in the bars. Results were analyzed for statistical significance using a one-way ANOVA, *P* < 0.04. Letters indicate significance in a two-tailed *t*-test, *P* < 0.01. (d) Typical images of *B. cinerea*-infected leaves of plants whose roots were pretreated with the indicated *T. virens* strains. (e) *X. euvesicatoria*: 5- to 7-week-old tomato plants were soil drenched with a spore suspension of the indicated *T. virens* genotypes (10^7^ spores mL^−1^) twice, then inoculated with 10^5^ CFU mL^−1^ of *Xcv* 2 h after the second drench. Plants treated with water were used as mock. 7 days after inoculation, leaf tissue was harvested, and the *Xcv* CFU/mg tissue was measured. Graph represents the results of 3 independent experiments ± SE, N = 9 for each treatment. Different letters indicate statistical significance using a one-way ANOVA with a Dunnett’s post hoc test, *P* = 0.0007.

Interactions of beneficial microorganisms with roots induces systemic defense responses. We refer to these collectively as “ISR” (induced systemic resistance [5, 6, 21]) here, although the biochemical basis for the plant’s response is more complex. The Q-WT strain did not promote growth under our conditions; however, the interaction of this strain with the roots of tomato seedlings had a strong protective effect against *B. cinerea* and *X. euvesicatoria* (Fig. 2). *T. virens* treatment of tomato roots provided ISR against leaf infection by the necrotroph, *B. cinerea* (Fig. 2). *ΔgliP* provided no significant protection, the phenotype was almost fully restored in the complemented strain, and the P-strain provided an intermediate level of protection (evident in terms of disease progression and severity [Fig. 2b and c], although not in lesion area [Fig. 2a]). The ability of the P-strain to confer an intermediate level of protection shows that in the absence of gliotoxin, other factors from the fungal partner promote ISR. In the tomato ISR assay used here, when challenged with the bacterial pathogen, *X. euvesicatoria*, the Q strain provided protection, while *ΔgliP* and the P-strain did not (Fig. 2e).

### Gliotoxin-producing competence increases plant immune responses.

**FIG 3:**
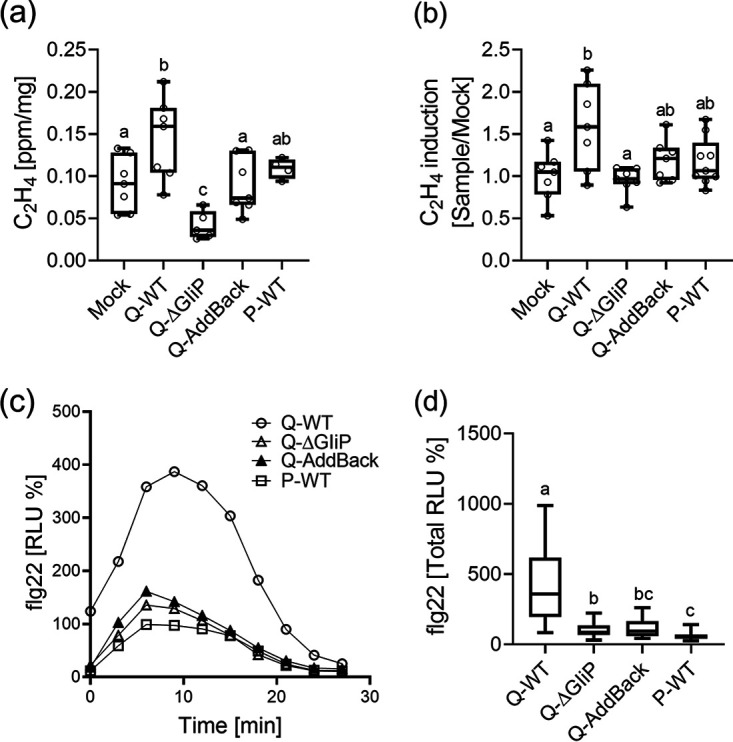
Plant immune responses to gliotoxin producer and nonproducer *T. virens* strains. 5- to 7-week-old tomato (MM) plants were soil drenched with a spore suspension of the indicated *T. virens* genotypes (10^7^ spores mL^−1^) twice. (a-b) Leaf disks were harvested from the fourth to fifth leaves, 4 h after the second drench, and sealed in glass vials. (a) Wounding ethylene and (b) ethylene production in response to the ETI elicitor EIX (1 μg/mL) were measured using gas chromatography after 4 h. Average ± SEM of 3 independent experiments is presented, N = 7. Results were analyzed for statistical significance using a one-way ANOVA, *P* < 0.006, with Tukey’s post hoc test (a: *P* = 0.0006; b, *P* = 0.012). Letters represent statistical significance in a two-tailed *t*-test, *P* < 0.03. Boxplots are shown with the interquartile ranges (boxes), medians (black lines in boxes), and outer quartile whiskers, minimum to maximum values. (c-d) Leaf disks were harvested from the fourth to fifth leaves, 24 h after the second drench, and placed in 96-well reflective plates. ROS production in response to the bacterial elicitor flg22 was measured immediately, for 25 min. (c) Time course of ROS burst. (d) Total ROS produced by each sample. Average ± SEM is presented for 3 independent experiments, N = 24. The peak (c) or total (d) RLU generated by the P strain (CABI) set as 100%. Letters represent statistical significance in a one-way ANOVA with a Dunnett’s post hoc test, *P* < 0.0001. Boxplots are shown with the interquartile ranges (boxes), medians (black lines in boxes), and outer quartile whiskers, minimum to maximum values.

To test whether the mechanism of protection involves the priming of resistance, we performed several assays that were independent of the foliar pathogen. Ethylene production in response to wounding (Fig. 3a) or the ETI elicitor, EIX (Fig. 3b), were higher in plants treated with the Q strain but not in those treated with its gliotoxin-deficient mutant. Wounding ethylene in *ΔgliP*-treated plants was actually lower than the “mock” control level, while expression of A. fumigatus *GliP* in *ΔgliP* restored the plant response to the control level (Fig. 3b). The mutant could not increase EIX-induced ethylene production, while the addback and P strains showed an increasing trend, though this overlapped statistically with both the control and Q strain-induced levels (Fig. 3b). Combined, these data suggest a more complex dependence of wound-induced ethylene production on whether the plant is interacting with *Trichoderma*, with or without gliotoxin production. In a second assay, flagellin-induced reactive oxygen species (ROS) production, the Q strain was again most effective, causing a striking increase in the ROS response to flagellin (Fig. 3c and d).

### Gliotoxin impact on the tomato transcriptome in response to *Trichoderma*.

**FIG 4:**
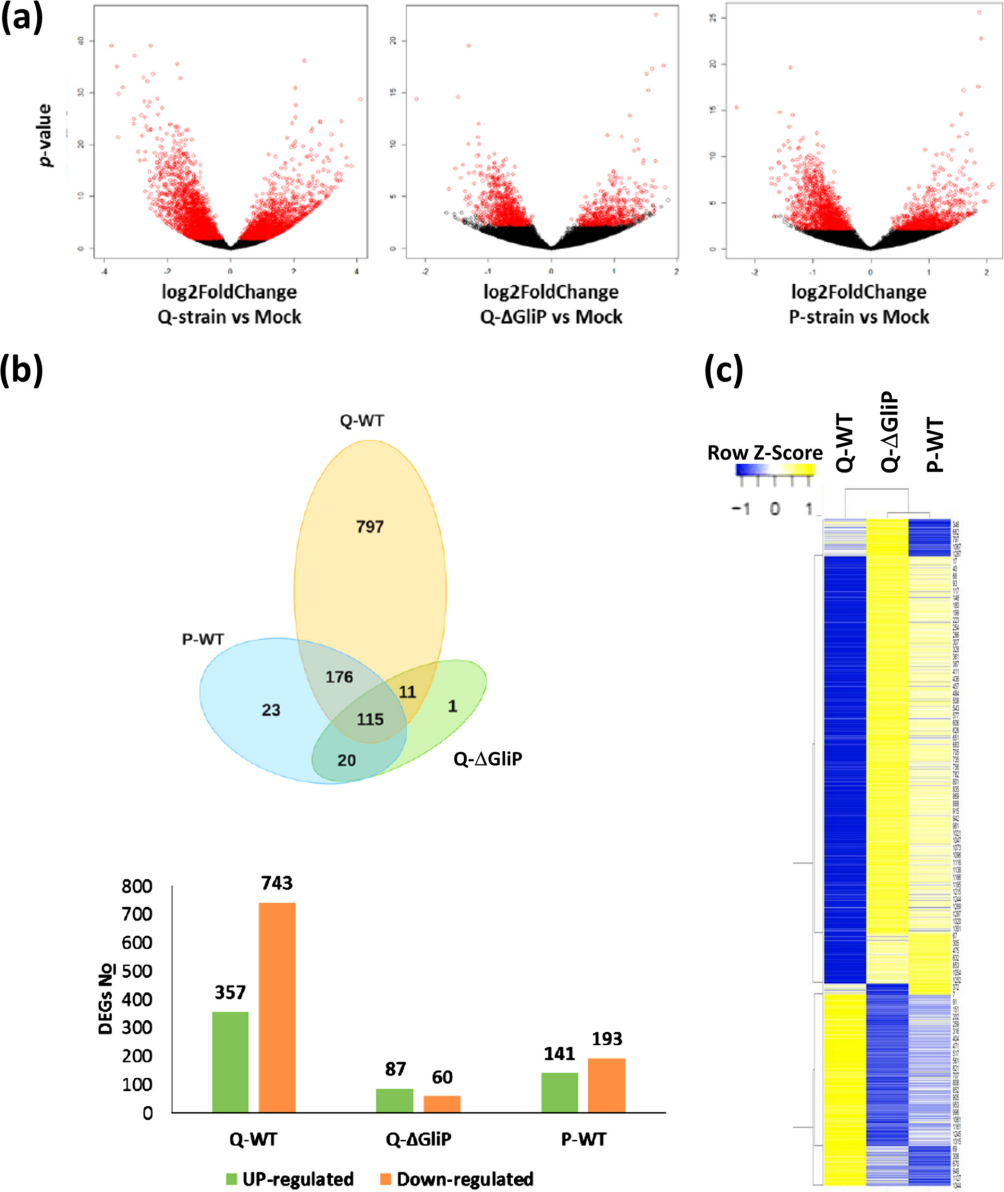
Overall impact of gliotoxin-producing and nonproducing *T. virens* strains on the pattern of differential gene expression in tomato shoots. (a) Volcano plots generated from the RNASeq data set. From left to right: Q-WT, Q-*ΔgliP*, and P-WT treated plants versus mock (sterile deionized water) controls. Black dots indicate genes whose expression levels were not significantly affected by *T.virens*, and red dots indicate DEGs that had significantly different expression levels compared to the untreated controls, with *P* < 0.05. (b) Venn diagram of total significant (*P* < 0.05) DEGs after a correction for the batch effect and the application of a >2-fold change cutoff. Number of upregulated and downregulated DEGs following each treatment, compared to untreated controls. (c) Expression heat map of the entire set of DEGs. Gene lists were constructed based on significant (*P* < 0.05) DEGs, with a cutoff of at least a 2-fold change following *T. virens* treatment of plant roots in at least one treatment group. Colors are assigned based on standard score (*z*-score), reflecting distance from the mean. The *z*-score of each gene was calculated in accordance with the log_2_-fold change of each DEG and treatment from the DEG list (Data set S2), which was corrected for the batch effect. The expression heat maps were constructed using the web-based http://heatmapper.ca utility (77) with the Spearman’s rank correlation method.

In view of the profound effects of gliotoxin production on the *Trichoderma*-plant interaction, we sought to develop a better understanding of the role this fungal metabolite has on the response of the plant to root colonization by the fungus. We performed a cell expression by linear amplification and sequencing (CEL-Seq) analysis of RNA samples extracted from tomato seedling shoots, 4 dpi, with our T. virens strain panel (Q-WT, Q*-Δglip*, and P-WT; *n* = 4 biological repeats per each treatment). Validation of RNASeq results by qPCR for three regulated genes is given in Fig. S1. The complete data set is provided in Data Set S1. The total numbers of genes whose expressions differed significantly from the mock treatment in each strain as well as comparisons between each pair of *Trichoderma* treatments are given in Table S2. Out of the three T. virens root treatments, the gliotoxin producing strain, Q-WT, had the most robust impact on the number of genes differentially expressed in the plant shoot compared to untreated control plants. Root treatments with T. virens strains that do not produce gliotoxin, the P-strain and Q-*ΔgliP*, affected gene expression, from the point of view of the number of differentially expressed genes (DEGs) detected, ~5-fold weaker than the Q-WT strain. Moreover, the P-WT and Q-*ΔgliP* did not differ in the number of DEGs compared to one another (Table S2). The same trends are evident in volcano plots (Fig. 4a).

10.1128/mbio.00389-22.2 TABLE S2 Pairwise comparisons of numbers of differentially expressed genes (DEG).12-day-old tomato seedlings were grown in ½ MS-agar magenta boxes and treated with spore suspensions of 100K spores/magenta dripped on the agar surface. The following *T. virens* strains were tested: Gv29-8 (Q-WT), gliotoxin deficient mutant in the same genetic background (Q*-ΔgliP*), and *T. virens* IMI 304061 (P-WT), or with sterile water as control (mock). 4 dpi, RNA samples (N = 4 for each treatment) were extracted from plant shoots and analyzed using cell expression by linear amplification and sequencing (CEL-Seq). The table summarizes overall differences between each of the indicated pairs of treatments. Total Number Genes: all the genes present in the annotation file used. All Zero: transcripts that were not detected in any of the samples. Low counts: genes which did not pass a minimal detection threshold. Tested: genes that are included in the statistical analysis and received a final adjusted *P*-value. Significant Up/Down: significantly DEG, according to a threshold of 0.05 on the adjusted *P*-value (separated into upregulated and downregulated, according to the sign on the log_2_-fold change) (Data set S1). Download Table S2, DOCX file, 0.1 MB. Copyright © 2022 Zaid et al. 2022 Zaid et al. https://creativecommons.org/licenses/by/4.0/ This content is distributed under the terms of the Creative Commons Attribution 4.0 International license.

10.1128/mbio.00389-22.3 DATA SET S1 Complete transcriptomic data. Download Data Set S1, XLSX file, 15.5 MB. Copyright © 2022 Zaid et al. 2022 Zaid et al. https://creativecommons.org/licenses/by/4.0/ This content is distributed under the terms of the Creative Commons Attribution 4.0 International license.

10.1128/mbio.00389-22.6 FIG S1 Validation of RNASeq quantitation by qPCR. Points indicate the signal measured by the two methods for the same treatments. The qPCR signal is 2^-ΔCt^ (signal relative to the housekeeping gene, UB-3) in the same sample. The dotted line is a power law trendline fit to the log-log plot of the combined data for the three genes. Download FIG S1, TIF file, 0.8 MB. Copyright © 2022 Zaid et al. 2022 Zaid et al. https://creativecommons.org/licenses/by/4.0/ This content is distributed under the terms of the Creative Commons Attribution 4.0 International license.

10.1128/mbio.00389-22.4 DATA SET S2 Complete DEGs list (filtered for batch effect, see main text). Download Data Set S2, XLSX file, 0.3 MB. Copyright © 2022 Zaid et al. 2022 Zaid et al. https://creativecommons.org/licenses/by/4.0/ This content is distributed under the terms of the Creative Commons Attribution 4.0 International license.

The fungal competence to alter the transcriptome of the plant was thus greater for the Q-WT strain, which affected the expression of more genes in a stronger manner than the two gliotoxin nonproducing strains, mutant Q-*ΔgliP* and the P-WT strain. This is clear in the volcano plot analysis, both in terms of the number of DEGs and in the magnitude of their change compared to untreated control plants, and in a Venn diagram and overall DEG counts (Fig. 4a and b). A cluster analysis (Fig. 4c) likewise shows a strikingly different DEG pattern between Q-WT and the nonproducing strains. The Q-*ΔgliP* and P-WT DEG clusters more closely resemble each other, but they are not identical. Most genes that were differentially expressed in tomato leaves following T. virens root treatment clustered into two main groups (Fig. 4c). The first group includes genes that were strongly upregulated by Q-WT and either weakly downregulated or unaffected by Q-*ΔgliP* and P-WT. The second group includes genes that were strongly downregulated by Q-WT and either weakly upregulated or not affected by Q-*ΔgliP* and P-WT. A principal component analysis (PCA) revealed that the biological samples that were analyzed from two different experimental batches, obtained some months apart, differed (Fig. S2). Specifically, the Q-WT samples were separated according to experimental batch in both principal components. One experiment from the mock treatments deviated downwards in PC2, which accounted for 18% of the variance, while the data for the two gliotoxin nonproducing strains showed small (P-WT) or apparently batch-independent (Q*-ΔgliP*) variation. As the batch effect was largest for the gliotoxin-producing strain (Q-WT), which is our main focus, we based the functional analysis on a more concise gene list, obtained after filtering to retain only those transcripts significantly regulated in both experimental batches individually (Data Set S2). Examining the ratios of the numbers of DEGs between treatments, similar proportions to those reached in the general results (Fig. S2) are seen in the filtered, reduced DEG list, but they were somewhat different between upregulated and downregulated DEGs within the same treatment (Fig. 4a, Fig. S3).

10.1128/mbio.00389-22.7 FIG S2 PCA plot demonstrating grouping by treatment and *Trichoderma* strain. Note the differences among the two experimental batches, primarily in PC2, which accounts for 18% of the variance. Fungal treatments are indicated by color (red, mock inoculated controls; green, Q-WT; blue, Q-*ΔgliP*; and purple, P-WT), with shape indicating the batch (circle for batch number 1 and triangle for batch number 2). The yellow dashed line emphasizes the distinct grouping of all Q-WT samples, where 54% variance is represented by PC1. Download FIG S2, TIF file, 0.7 MB. Copyright © 2022 Zaid et al. 2022 Zaid et al. https://creativecommons.org/licenses/by/4.0/ This content is distributed under the terms of the Creative Commons Attribution 4.0 International license.

10.1128/mbio.00389-22.8 FIG S3 DEGs counted from the complete gene list. (a) Venn diagram of total significant (*P* < 0.05) DEGs, following at least one of the *T. virens* treatments, after applying a >2-fold change cutoff. (b) Number (DEG No) of significantly upregulated and downregulated tomato genes for each *T. virens* strain, compared to untreated controls. Download FIG S3, TIF file, 0.6 MB. Copyright © 2022 Zaid et al. 2022 Zaid et al. https://creativecommons.org/licenses/by/4.0/ This content is distributed under the terms of the Creative Commons Attribution 4.0 International license.

### Functional analysis of the tomato leaf transcriptome in response to *T. virens*.

**FIG 5:**
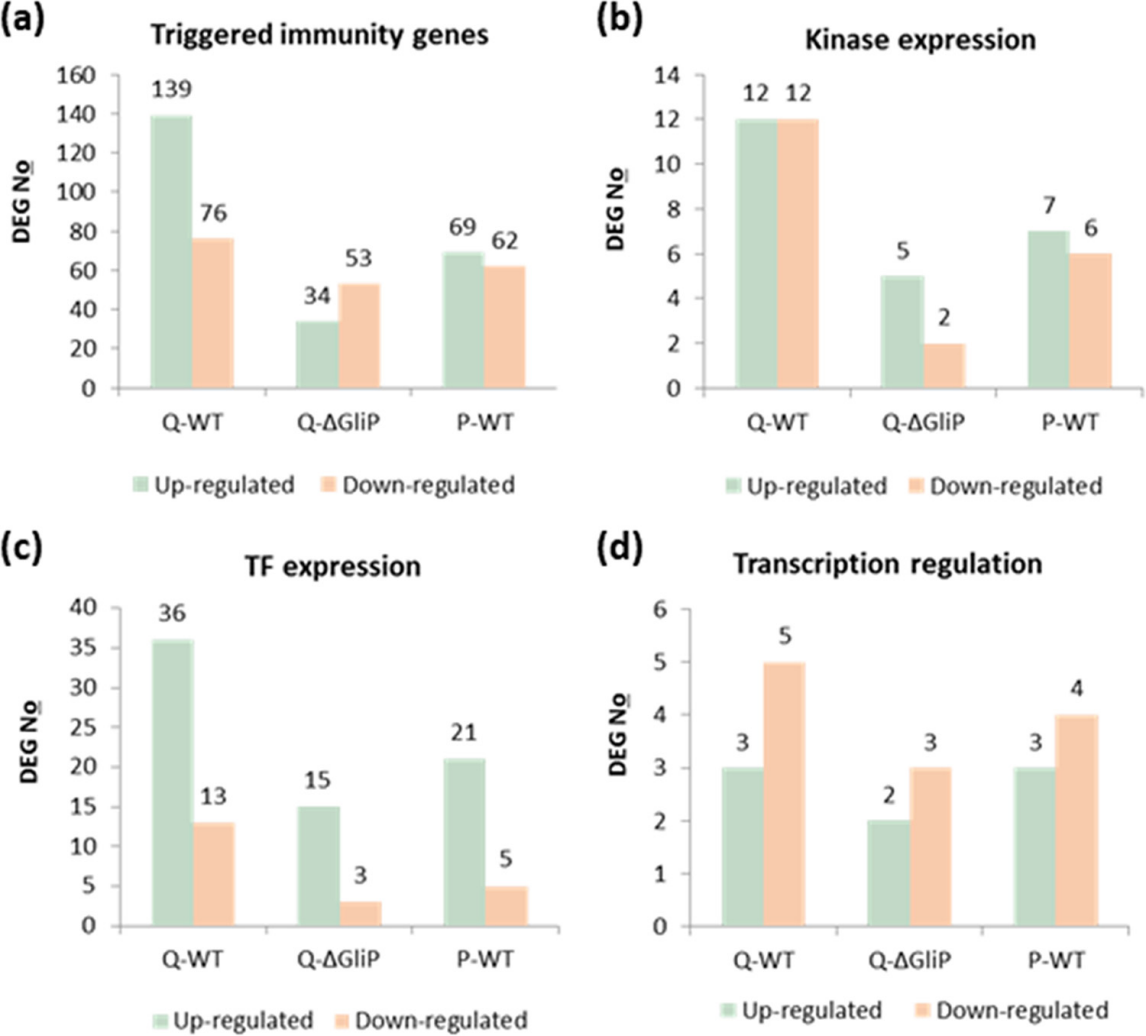
Functional annotation classes. Significant number of regulated DEGs following each *T. virens* treatment. Genes were counted according to pre-prepared gene lists and were considered regulated DEGs with a cutoff of at least a 2-fold change. Upregulated DEGs are indicated in green, and downregulated DEGs are indicated in orange. The *T. virens* strain is indicated under the bars. (a) Triggered immunity-related genes, based on a list compiled from (78–80), and three more analyses based on gene lists retrieved from the iTAK (45) database; (b) kinases; (c) transcription factors; and (d) transcription regulation.

Next, we wanted to explore the functional aspects of these transcriptomic alterations. As a first step, we counted DEGs in each T. virens treatment, according to four annotated gene categories (Fig. 5a–d): 1) triggered immunity (induced immune response) related genes, giving an indication of the plant defense response to T. virens treatments; 2) plant kinase expression, giving an indication of modification in signaling activity in response to T. virens treatments; 3) transcription factors (TF), giving an indication of alterations in gene expression; and 4) transcription regulation, giving an indication, together with category 3, of a shift in gene transcriptional patterns. These annotations are from the iTAK database (45).

According to the DEG number analysis, the T. virens gliotoxin-producing Q-strain had the strongest effect on the expression of plant genes whose annotations are linked to an induced immune response, mainly over the upregulated DEGs (Fig. 5a), with its gliotoxin deficient mutant affecting only 24.5% and the P-strain affecting 49.6% of the upregulated DEGs. Both gliotoxin nonproducing strains had a similar effect on the downregulated DEG number, impacting ~70% genes compared to Q-WT, indicating that most of the DEGs influenced by gliotoxin were upregulated genes. Kinase expression was also affected most strongly by Q-WT treatment, affecting 24 kinase genes (upregulated or downregulated), with P-WT impacting 13 and Q-ΔgliP impacting 7. In all groups, there were similar numbers of upregulated and downregulated differentially expressed kinases (Fig. 5b). TF expression was similarly affected by the three treatments (Fig. 5c). Nonetheless, the effect of T. virens treatments on transcription regulation (Fig. 5d), as opposed to transcription factors (Fig. 5c), was similar with all three strains, suggesting that global transcription regulators are not strongly affected by gliotoxin.

Among the DEGs markedly upregulated following Q-WT treatment were genes related to the plant response to stresses and to the hormone response. A few examples are: a NINJA family gene (JA pathway, Solyc04g005380), 1-aminocyclopropane-1-carboxylic acid oxidase (ethylene biosynthesis, Solyc07g049530), endochitinase (Solyc02g082920), osmotin-like (Solyc08g080650), and WRKY transcription factor (Solyc08g082110) (Data Set S3). In contrast, Q-ΔgliP and P-WT hardly altered the expression of these genes (Data Set S3).

10.1128/mbio.00389-22.5 DATA SET S3 Growth- and defense-related DEGs (manually curated list with annotations). Download Data Set S3, XLSX file, 0.8 MB. Copyright © 2022 Zaid et al. 2022 Zaid et al. https://creativecommons.org/licenses/by/4.0/ This content is distributed under the terms of the Creative Commons Attribution 4.0 International license.

**FIG 6:**
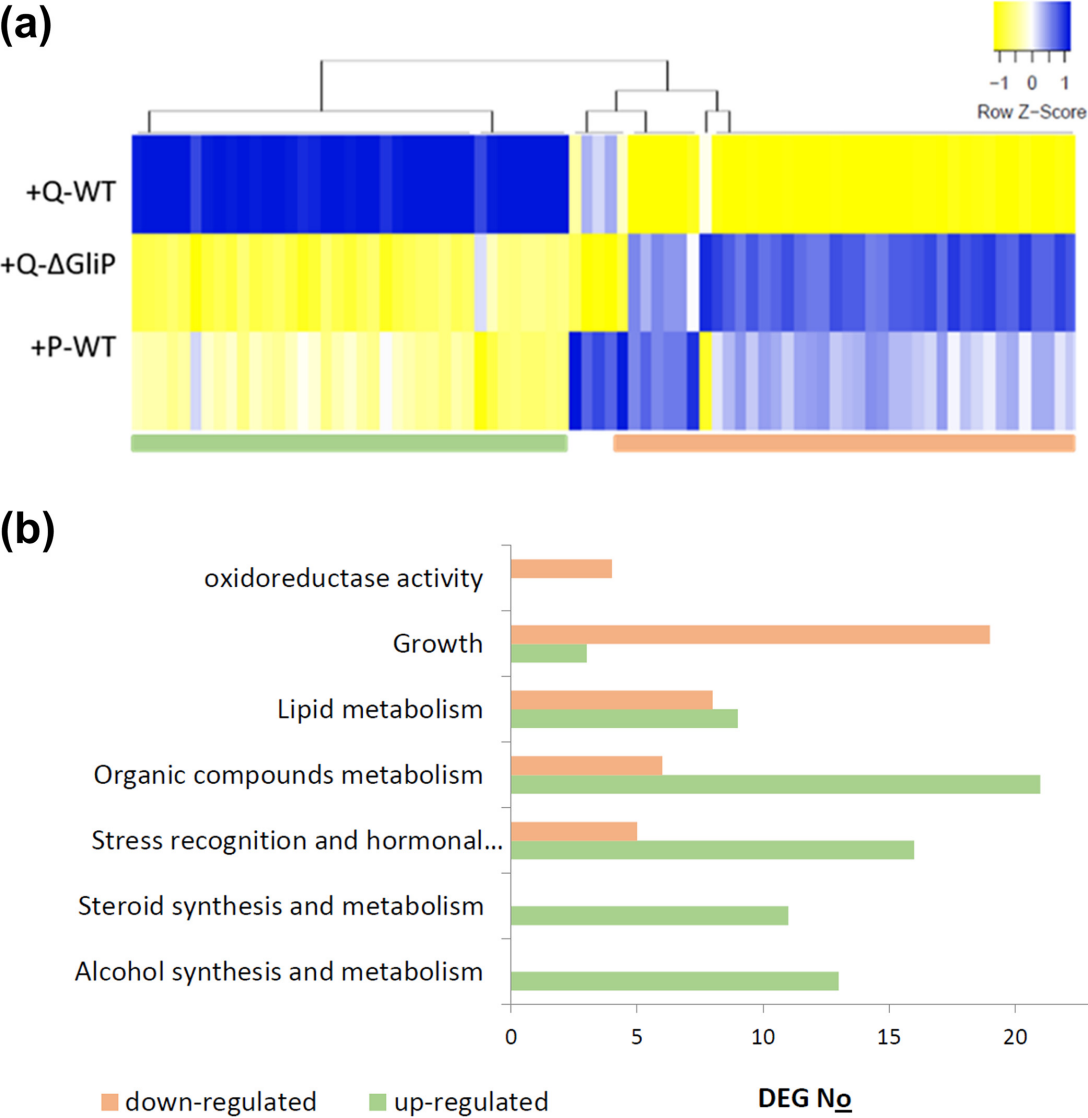
Functional analysis. (a) Cluster analysis: expression heat maps were constructed using the web-based http://heatmapper.ca utility (77) with the Spearman’s rank correlation method, based on a manually curated list of 80 genes, with annotations related to defense and growth (Data set S3), that are regulated by the Q-WT strain and were extracted from the list of significant (*P* < 0.05) DEGs (Data set S2). The *z*-scores per gene were calculated from the log_2_-fold change of each DEG and treatment from the DEG list (Data set S2), which was corrected for the batch effect. (b) Functional annotation. The two clusters from (a) were analyzed separately with a biological network server (GeneMANIA, [81]). The server accesses annotation for Arabidopsis thaliana, so the Arabidopsis orthologs of the tomato genes were matched before doing the calculation (using a list kindly provided by the Lifschitz lab [82]). The graph shows the number of annotation subnetworks generated by the server, rather than the gene count, so that if a gene appears in more than one subnetwork, it is counted according to the number of subnetworks.

To further test the impact of gliotoxin on functional enrichment, using a different strategy, we created a manually curated list of 80 genes (Data Set S3) related to plant defense and growth regulation, considering that there are tradeoffs between the two. Based on this list, we constructed a heat map, showing that most of the DEGs in this subset clustered into two groups: genes that are upregulated following root treatment with Q-WT but not significantly changed or downregulated in the gliotoxin-lacking Q-*ΔgliP* mutant and the P-WT strain, and vice versa (Fig. 6). As evident from Fig. 6b, genes whose annotation suggests a relation to growth and development are downregulated by T.virens gliotoxin competence. In contrast, annotations related to stress and hormone response correspond to more of the upregulated genes. These results indicate that gliotoxin not only has a strong impact on gene expression in terms of the number of genes whose expression is affected but also directs the plant toward defense-oriented regulation.

### Direct effect of gliotoxin on the plant immune response.

**FIG 7:**
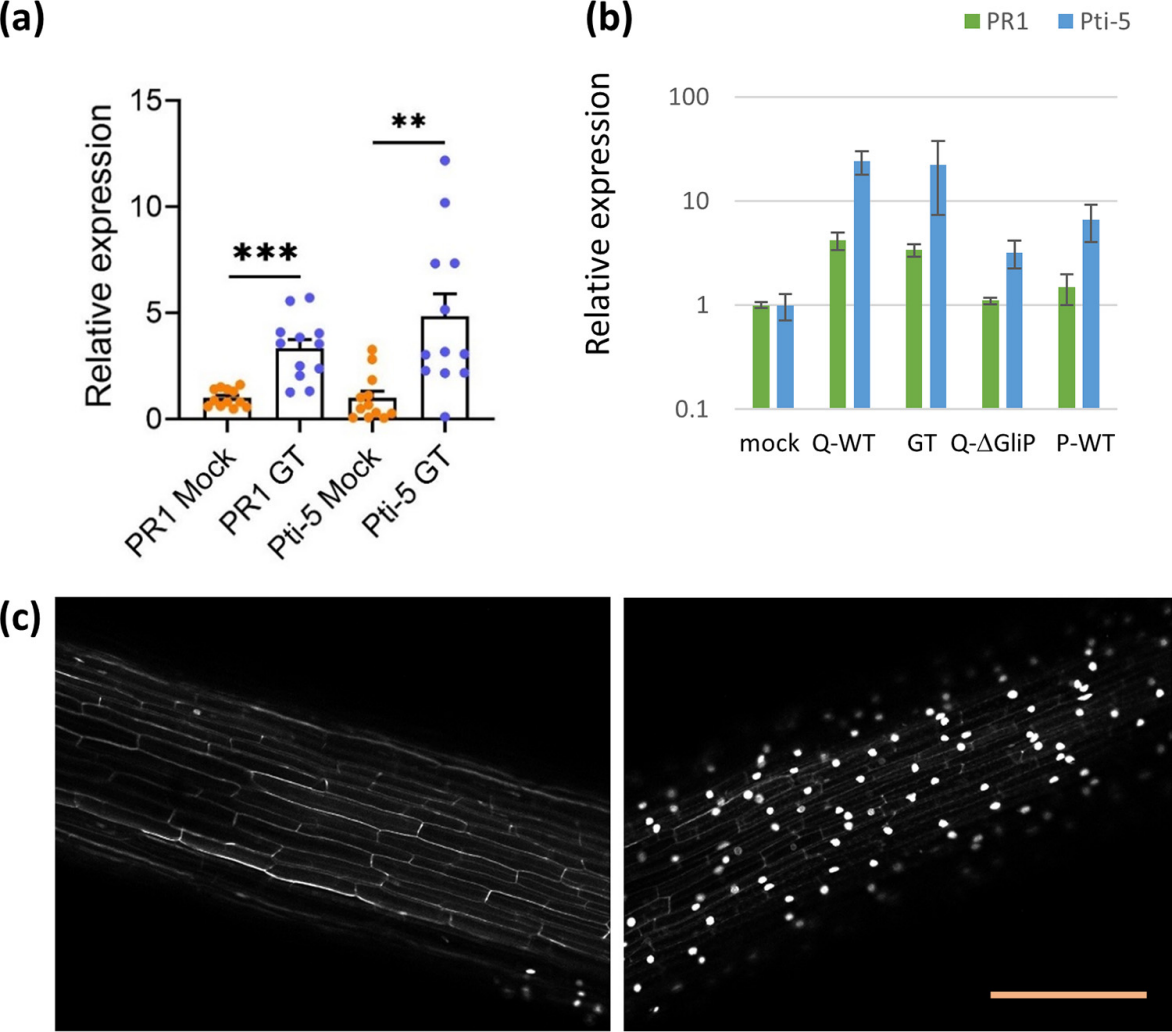
Induction of defense gene expression in response to gliotoxin treatment. (a) Expression of two defense genes, PR1 and Pti-5, in tomato cotyledons, in response to gliotoxin (GT) added to the growth medium. (b) Expression of PR1 and Pti-5 in shoots of seedlings treated with Q, *ΔgliP*, and P strains, compared to data for gliotoxin which are replotted from (a). Bars indicate SEM for 4 biological repeats, except for the GT experiment for which the individual points are plotted in boxplot format in panel (a). Expression levels were assayed by qPCR, relative to UB-3 as a housekeeping gene, and normalized to the average value for the mock (water) control. Tomato seedlings were grown as in Fig. 1. (c) Root cell death following exposure to gliotoxin. Propidium iodide staining of nuclei is visible in confocal images of root epidermal and cortex cells of the primary roots of tomato plants grown in hydroponic culture: left, mock; right, GT-treated; scale bar, 200 μm.

Interactions with producing (Q) and nonproducing (*ΔgliP* and P strain) T. virens strains resulted in strikingly different gene expression profiles in the host plants. To address the question of whether this difference can be attributed in part to the direct perception of gliotoxin, we investigated whether the purified metabolite can induce expression of plant defense-related genes. The overall pattern and extent of the induction of PR-1 and Pti-5 expression were qualitatively similar for pure gliotoxin (Fig. 7a) and for interaction with the Q strain (Fig. 7). PR-1 was induced by the Q strain or gliotoxin, while Pti-5 also responded to the *ΔgliP* mutant and the P strain, though somewhat less than it did to the Q-WT strain. The P and Q strains, or GT applied to the agar surface, cause a variable extent of cell death. To confirm the phytotoxicity of GT, the metabolite was applied uniformly in semihydroponic culture. At a GT concentration of 60 μg/mL which is representative of that produced by the Q strain in the soil (46, 47), propidium iodide stained a majority of epidermis and outer cortex cells (Fig. 7c), while the controls showed scattered staining, often associated with root hairs (Fig. 7c). Furthermore, gliotoxin had direct effects on ROS production and ion leakage from leaf disks (Fig. S4).

10.1128/mbio.00389-22.9 FIG S4 Direct effect of gliotoxin on ion leakage and ROS production from leaf disks. Leaf disks were harvested from 5-week-old Moneymaker plants. (a) For conductivity measurements, disks were washed in DDW for 4 h and sealed in glass vials with either 100 μg/mL gliotoxin or an identical volume of DMSO (mock). Conductivity in the samples was measured after 48 h of gentle shaking (75 rpm). (b) For total ROS production, leaf disks were placed in 96-well reflective plates and washed for 2 h in DDW. The water was then removed and replaced with either an HRP-luminol reaction mixture that contained 100 μg/mL gliotoxin, or an identical volume of DMSO (mock). ROS production was measured immediately for 30 minutes. Both experiments were conducted 3 independent times. Asterisks denote a significant increase in conductivity and ROS with gliotoxin treatment in a *t*-test with Welch's correction. A: N = 18, *P* < 0.0001. B: N = 40, *P* < 0.01. Download FIG S4, TIF file, 0.7 MB. Copyright © 2022 Zaid et al. 2022 Zaid et al. https://creativecommons.org/licenses/by/4.0/ This content is distributed under the terms of the Creative Commons Attribution 4.0 International license.

### Priming of plant defense by gliotoxin.

**FIG 8:**
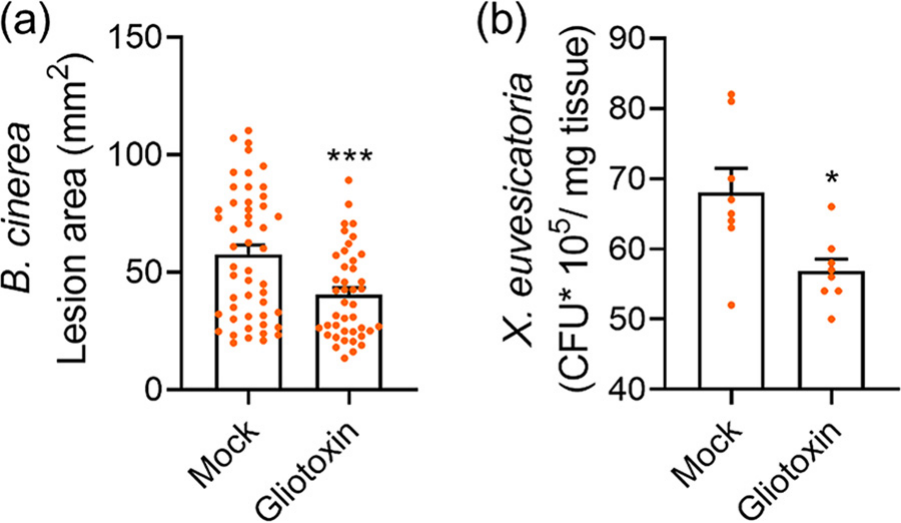
Priming of defense by exposure to gliotoxin. (a) 5-week-old tomato MM plants were pre‐treated with gliotoxin (60 μg/mL, 5 mL/plant, applied via soil drench), or mock‐treated with an equal volume of DMSO in water (1:1667 of DMSO), then infected with *B. cinerea* 3 days after gliotoxin treatment, as described in the text. The experiment was repeated 3 times, N = 42. Asterisks denote a significant decrease in gray mold disease with gliotoxin pre‐treatment in a *t*‐test with Welch's correction, ***, *P* < 0.001. (b) Plants were grown as in (a), then inoculated with *X. euvesicatoria* 3 days after gliotoxin treatment, as described in the text. The experiment was repeated twice, total N = 8. Asterisks denote a significant decrease in *X. euvesicatoria* CFU in plants pre‐treated with gliotoxin in a *t*‐test with Welch's correction, *, *P* < 0.05.

According to the genetic evidence in Fig. 2, gliotoxin contributes a major part of the priming of tomato plants by the T. virens Q strain for resistance to both pathogens studied. This was tested directly by replacing *Trichoderma* with pure gliotoxin (Fig. 8). The treatment of tomato plants with gliotoxin by soil drench replaced, to a great extent, interaction with the T. virens Q strain.

## DISCUSSION

The generally beneficial interactions of *Trichoderma* spp. with plants include directly antagonizing pathogens in the soil and priming, through their interaction with roots, for systemic resistance to infection by foliar and soil-borne pathogens. Here, we addressed the role of the secondary (specialized) metabolite (SM) gliotoxin in one node of the three-way plant-*Trichoderma*-pathogen interaction. GT produced by some T. virens strains can facilitate the antagonism of pathogens, but it is also reportedly phytotoxic in some plants. This tradeoff is a factor considered in the development of biocontrol strains (1). In an evolutionary perspective, our results suggest a species/strain specific role of GT in T. virens interactions with plant roots, analogous to the one studied for mycoparasitism (38). The P strain is not simply identical to a gliotoxin-deficient mutant of a Q strain; the P strain lacks the gliotoxin biosynthesis cluster, but it produces a related metabolite, gliovirin. Furthermore, there are additional sequence differences between the genomes (48, 49). Overall, comparison of the Q strain with its GT-deficient mutant and a GT nonproducing P strain (Fig. 2) showed that the metabolite provides a major contribution to the plant’s ISR response. This is supported by the transcriptomic signature of these strains, with the Q strain upregulating genes belonging to a list compiled from annotations related to plant defense (Data Set S2). As found for the antagonism of fungi (38), a strain that is not a GT producer could promote immune priming and systemic resistance by GT-independent pathways. Indeed, root inoculation with the same P strain studied here gave a 2- to 3-fold protection, relative to controls without *Trichoderma*, to cucumber seedlings against Pseudomonas syringae pv. *lachrymans*. In greenhouse assays, it provided control of two soil-borne fungal pathogens, Rhizoctonia solani and Sclerotium rolfsii, in bean (50). Furthermore, this same P strain is known to suppress *Pythium* sp., R. solani, and S. rolfsii (51–53). Nevertheless, when the wild-type *Trichoderma* strain is a GT producer, this metabolite takes a dominant (though not exclusive) role (Fig. 2). In the tomato-T. virens interaction, we have therefore defined gliotoxin as a small-molecule inducer of systemic plant immune responses (ISR and other overlapping pathways) and defense gene expression. Pure GT induces plant defense gene expression (Fig. 7) and confers significant protection against infection by two pathogens (Fig. 8). T. virens Gv29-8 does not promote growth under some laboratory conditions (Fig. 1) (41). The same isolate caused a browning, apparently stress-like, response in maize roots (54). In contrast, when inoculated at a distance from the roots, Gv29-8 promotes growth (39). T. virens cannot be considered a pathogen, as it is not adapted to invade the host beyond the outer root layers, nor does it generally cause any disease symptoms. This strain induces a strong ISR response and controls soil-borne cotton seedling diseases when applied as a seed treatment (55, 56). Likewise, overproduction of the terpene HA (and consequently gliotoxin, viridin, and viridiol) in Gv29-8 (resulting from loss of the NRPS Tex7) slowed growth in maize, yet it did not affect the potential of Gv29-8 to suppress southern corn leaf blight (35).

The demarcation between symbiont and pathogen is not perfectly sharp. Pathogens can prime the plant immune system against subsequent infection (for example, the SAR response). In the pathogen context, fungal SM act as toxins (57). Sirodesmin, an ETP toxin like GT, is a virulence factor for L. maculans (58). Gliotoxin is a virulence factor for A. fumigatus, an opportunistic human pathogen (59). Indeed, GT produced by A. fumigatus in systemic infection is immunosuppressive. In a mouse model, pretreatment with GT raised susceptibility to systemic infection (60). GT was reported to be phytotoxic in a simple germination test (61). Similarly, GT from A. fumigatus was inhibitory to lettuce seedling growth (62). GT inhibited the growth of cultured tobacco cells and seedling roots via interference with the production of branched-chain amino acids by inhibiting acetolactate synthase (63). It is known that gliotoxin inhibits the production of enzymes by some plants and negatively affects vegetative growth by inhibiting growth and seed germination (1, 63–65).

All these effects of GT would predict a negative outcome for plants interacting with GT-producing *Trichoderma* strains. On the contrary, the Q strain effectively primed plant immunity, while its GT-lacking mutant *ΔgliP* was much less efficient (Fig. 2). Thus, production of the same compound is encoded in orthologous gene clusters in two distantly related fungi: in the opportunistic human pathogen, A. fumigatus, GT suppresses immunity, while in the opportunistic plant symbiont, Trichoderma virens, it induces plant defense. Although full biochemical mechanisms can never be inferred from transcriptomics alone, our data indicate that gliotoxin could act as a MAMP, triggering an immune response in plants. Limited root damage noticed in gliotoxin-producing strains, however, could also release DAMP signals. In this context, we note that localized root cell death is observed in P. indica interactions (66, 67), though this symbiont does not produce known phytotoxic SM. Several mechanisms could be acting here in parallel, with MAMP, DAMP, and even effector-like activities overlapping, as for plant-pathogen interactions (24). Field use of a toxic molecule might not be the best for agriculture, even though GT is easily degraded. Nevertheless, the concept of a fungal small-molecule inducer replacing, at least in part, interaction with the fungus is an interesting one. Pure gliotoxin, apparently by promoting limited cell death or by additional mechanisms, induced some defense responses and systemic resistance in the absence of *Trichoderma* interaction with the roots (Fig. 7 and 8). Thus, the separation of microbe-associated (MAMP) and damage-mediated (cell death or DAMP) mechanisms is worthy of further study. From the agricultural point of view, it is important to consider that the outcome of the *Trichoderma*-plant interaction depends on plant growth stage, age, cultivar, fungal strain, and environment. We have consistently observed significant growth promotion by the Q-WT strain Gv29-8 over longer growth periods (unpublished observations). Field and greenhouse productions of GT-producing strains have been commercialized as plant disease biocontrol agents and as plant growth promoters (1). This obviously would not be possible, were the seedling effects observed here, in a contained artificial condition, to dominate the interaction of T. virens with plants in the field. Soil may provide a buffer, serving to limit gliotoxin delivery to plant roots, and GT is degraded faster at an alkaline pH, with the degradation also depending on soil microorganisms (47). Indeed, even at the seedling stage, Gv29-8 promoted both growth and lateral root formation when inoculated at a distance from the plants (44). Some *Trichoderma* SM are known to be inhibitory to roots at a high concentration but stimulatory at a lower concentration (34), thus showing a concentration optimum. Examples include harzianic acid (68) and 6-pentyl-2*H*-pyran-2-one (43). The potential of gliotoxin to cause limited cell death, could, nevertheless, be one component of the robust priming of the plant immune system, and it might facilitate root colonization and disease reduction under field conditions.

## MATERIALS AND METHODS

### Fungal strains and culture conditions.

All *Trichoderma* strains were grown in sterile conditions on potato dextrose agar (PDA, Difco) plates. Cultures were maintained in a controlled environment, at 22 to 25°C with a 16/8 h light-dark cycle. For long-term storage, a dense conidial suspension was suspended in liquid PDYC medium (24 g/L potato dextrose broth, 2g/L yeast extract, and 1.2 g casein hydrolysate; all from Difco), supplemented with 20% glycerol, for storage at −70°C.The sequenced reference strain (69, 70) Gv 29-8 (Q-WT) is deposited at the Fungal Genetics Stock Center (FGSC number 10586). The *ΔgliP* mutant (ΔGliP44-4) and the complemented strain (Q-Addback; *ΔGliP44* complemented with Aspergillus fumigatus *GliP*) were from the Kenerley lab (3). T. virens IMI 304061 (P-WT, lacking the gliotoxin biosynthetic cluster and producing, instead, gliovirin [48, 49]) is deposited at CABI, UK (https://www.cabi.org/).

### Plant materials and growth conditions.

Seeds of the Solanum lycopersicum L. cultivar Moneymaker (MM) were used throughout the study. Plants were grown from seeds in soil (Green Mix; Even‐Ari, Ashdod, Israel) in a growth chamber, under long day conditions (16 h:8 h, light:dark) at 24°C. For experiments performed in sterile conditions, seeds were surface sterilized by immersion in 1% sodium hypochlorite in sterile distilled water for 10 min, then washed in sterile water. Seeds were placed in plant culture “magenta” boxes (5 plants per box) or 23 × 23 cm square petri dishes containing sterile half-strength (0.5x) MS medium (71). For experiments with pure gliotoxin, the compound (Sigma) was applied to the agar surface around the seedling roots at 60 μg/mL (in water, from 10 mg/mL stock in DMSO), 1 mL onto 70 mL solid medium, at day 9 and again at day 10. Then, 24 h later (3 days total from the first application of gliotoxin), the cotyledons were harvested for RNA extraction. For microscopy of the gliotoxin-treated roots, seeds were germinated for 4 days, then transferred to nylon mesh overlaying liquid 0.5x MS medium, and the seedlings were cultured hydroponically with gentle rotary shaking for 2 days. Gliotoxin was added at day 2 to a final concentration of 60 μg/mL. After 2 days, further growth root sections were excised, stained with propidium iodide (10 μg/mL), and imaged. Control seedlings were mock-treated with water or with DMSO at the same concentration added from the gliotoxin stock solution. For pathogenesis and immunity assays conducted on gliotoxin-treated plants, 5-week-old MM plants were soil drenched with a final concentration of 60 μg/mL (5 mL/pot) gliotoxin in water. Mock treatments consisted of water with equal volumes of DMSO (1:1667 of DMSO). Plants were infected 3 days after GT treatment with B. cinerea or X. euvesicatoria, and lesion size and CFU were assayed for each pathogen, respectively, at 5 days for B. cinerea and at 7 days for X. euvesicatoria, as described in Fig. 2. For the immunity assays in Fig. S4, the tissue was harvested 3 days after gliotoxin treatment. For assaying the direct effect of gliotoxin on plant immunity (Fig. S4a), 100 μg/mL gliotoxin was added to tissues harvested from untreated 5-week-old MM plants.

### Trichoderma growth and treatments.

T. virens strains were maintained on potato dextrose agar (PDA) (Difco) plates and incubated at 22°C for 5 to 7 days. Plates were incubated in ambient light and temperature (300 μmol m^−2^ s^−1^, 25°C) to induce sporulation. Spores were collected 1 to 2 days later, suspended in distilled water, and filtered through cheese cloth or gauze to reduce mycelial fragments. Spore concentration was adjusted to 10^7^ spores mL^−1^ using a hemocytometer. 15 mL of the spore suspensions were applied to tomato plants by soil drench to the root system twice: 3 days before and 2 h before pathogen inoculation. For the magenta box cultures, 1 mL of 5 × 10^4^ spores/mL suspension was applied to the agar surface 3 days before pathogen infection or the harvest of leaf samples for RNA extraction. For large plates, 1 mL of 10^5^ spores/mL suspension per plate was applied to the agar surface by adding a few drops near each seed.

### Pathogen infection and disease monitoring.

Botrytis cinerea (Bc, isolate BcI16) was used for necrotrophic fungal disease monitoring. Cultures were maintained on potato dextrose agar (PDA) (Difco Lab) plates and incubated at 22°C for 5 to 7 days. B. cinerea spores were harvested from PDA plates in 1 mg mL^−1^ glucose and 1 mg mL^−1^ K_2_HPO_4_ and filtered through gauze. Spore concentration was adjusted to 10^6^ spores mL^−1^ using a hemocytometer. Tomato leaflets harvested from the fourth to fifth leaves of 5- to 7-week-old plants were detached from the plants 2 h after the second *Trichoderma* soil drench and inoculated with droplets of 10 μL spore suspension. Botrytis disease was found to be similar on whole plants and on detached leaves in several cases (21). Inoculated excised leaves were kept in a humid growth chamber at 22°C. Controls consisted of plants or leaves treated with water/buffer. The area of the necrotic lesions was measured after 5 to 7 days (as noted in the legend to Fig. 2) post-inoculation using ImageJ.

Xanthomonas euvesicatoria (*Xcv*, strain 85-10) was used for bacterial infection analysis. Bacterial cultures were grown in LB medium containing 100 mg L^−1^ of rifampicin and 300 mg L^−1^ of streptomycin overnight at 28°C. Bacterial cultures were centrifuged and re-suspended in 10 mM MgCl_2_ at a final concentration of 10^5^ CFU mL^−1^ (calibrated by OD_600_ measurement of a concentrated suspension). Fourth to fifth leaves from 5- to 7-week-old tomato plants were infiltrated in the abaxial side with the bacterial suspensions using a blunt end syringe. Seven days after infiltration, three leaf disks of 0.9 cm in diameter were sampled from at least four plants from each treatment, then ground in 1 mL of 10 mM MgCl_2_. Bacterial CFU was determined by plating 10 μL from 10-fold serial dilutions and counting the resulting colonies. Negative controls consisted of 10 mM MgCl_2_ without pathogen inoculation. For equal bacterial loading verification control, leaf disks harvested 4 h after infiltration were examined.

### Ethylene, reactive oxygen species (ROS), and ion leakage assays.

Assays of physiological reporters of plant immune response were conducted on leaf disks from plants treated as indicated. Ethylene production was measured as previously described (29). Leaf disks 0.9 cm in diameter were harvested from plants treated as indicated. Disks were washed in water for an hour. For each sample, six disks were sealed in a 15 mL glass tube containing 1 mL assay medium (with or without 1 μg mL^−1^ EIX) overnight. Ethylene production was measured by gas chromatography (Varian 3350, CA, USA). ROS were determined as previously described (29). Leaf disks of 0.5 cm in diameter were taken from the fourth to sixth leaves of 5 to 6-week-old plants. Disks were floated in a white 96-well plate (SPL Life Sciences, South Korea) containing 200 μL distilled water overnight at room temperature. After incubation, water was removed, and a ROS measurement reaction mixture containing either 1 μM flg-22 (Phytotechlabs, USA), 1 μg/mL EIX purified according to (72), or water (mock) was added. Light emission was immediately measured using a luminometer (Tecan Spark, Switzerland). Ion leakage from leaf disks was followed as described (29). Leaf disks (0.9 cm diameter) were harvested from 5-week-old plants and washed with water in a 50 mL water tube for 3h. For each sample, five leaf disks were floated in a 12-well plate containing 1 mL of water with or without 100 μg/mL gliotoxin (adaxial surface down) at room temperature with 100 rpm agitation. Controls of water alone and water with 100 μg/mL gliotoxin, without plant tissue, were also included. The net leakage after 48 h was measured with a conductivity meter (AZ Multiparameter pH/Mv/Cond./Temp Meter 86505, Taiwan).

### Statistical analyses.

All experimental data are presented as averages ± SEM in bar graphs or as minimum to maximum values in boxplots. Differences between two groups were analyzed for statistical significance using two-tailed *t*-tests with Welch's correction for unequal variances and the Holm-Sidak correction for multiple comparisons, where relevant. Differences among three groups or more were analyzed for statistical significance with a one-way ANOVA. Regular ANOVA was used for groups with equal variances, and Welch's ANOVA was used for groups with unequal variances. When a significant result for a group in an ANOVA was returned, the significance of the differences between the means of different samples in the group were assessed using a post hoc test. Tukey’s test was employed for samples with equal variances when the mean of each sample was compared to the mean of every other sample. Bonferroni’s test was employed for samples with equal variances when the mean of each sample was compared to the mean of a control sample. Dunnett’s test was employed for samples with unequal variances. Statistical analyses were conducted using Prism8.

### Microscopy.

Confocal root images were taken with the LSM 510 or LSM 700 axio-imager confocal microscope from Zeiss with a 25x Objective (LCI Plan-Neofluar with effective NA of 0.8). Excitation wavelength/fluorescence emission recording: 488 nm/525 nm for wheat-germ agglutinin-Alexa fluor-488 conjugate (Thermo Fisher Scientific) and 561 nm/595 nm for propidium iodide.

### RNA isolation.

Shoots of plants inoculated at day 10 and at day 12 were ground in liquid N_2_. The powder was re-suspended with TRI-reagent (T9424, Sigma), and RNA was further purified using the Direct-zol RNA miniPrep Kit (R2050, Zymo) according to the manufacturer’s guidelines. RNA was quantified by nanodrop or by Qubit Fluorometer (Invitrogen) using the Qubit RNA BR (Broad-Range) assay kit (Molecular probes, Q10211). RNA quality and integrity were evaluated by electrophoretic separation in the TapeStation system with RNA ScreenTape (Agilent).

### Real-time quantitative-PCR (RT-qPCR).

cDNA synthesis was performed using the qScript cDNA synthesis kit (Quanta-bio, 95047) according to the manufacturer’s guidelines. Abundance of transcripts was measured by RT-qPCRs performed in an Applied Biosystems 7000 cycler. Approximately 15 ng of cDNA were used as the template. The 15 μL reaction volume included 7.5 μL of 2x PerfeCTa SYBR green FastMix Low ROX (Quanta-bio, 95073) and 250 nM final concentration of specific primers for the gene of interest. Assays were run in triplicates, using the following thermal cycling protocol: initial denaturing at 95°C for 3 min; 40 cycles of 95°C for 10 s, 60°C for 45 s; followed by a gradual increase in temperature from 60°C to 95°C during the dissociation stage. Relative expression values were calculated using the comparative 2-^ΔΔCt^ method (73). The tomato genes and primers used are listed in Table S1. If defense gene levels in an untreated control sample were higher than those observed in interaction with the Q-WT strain, presumably indicating an undetected infection, the entire experiment was excluded from the analysis. Furthermore, outliers among triplicates within the same experiment, defined as observations differing by an order of magnitude but showing the same differential trends as the other samples in the group, were excluded.

10.1128/mbio.00389-22.1 TABLE S1 Primers used for RT-qPCR. Forward and reverse primers are from (83), except for the U-box pair, which is a standard pair used across many plant species. Download Table S1, DOCX file, 0.1 MB. Copyright © 2022 Zaid et al. 2022 Zaid et al. https://creativecommons.org/licenses/by/4.0/ This content is distributed under the terms of the Creative Commons Attribution 4.0 International license.

### Tomato shoot differential gene expression (DEG) profiling following *Trichoderma* root infection.

In order to estimate gliotoxin impact on tomato shoot transcriptome signatures, we tested DEG profiles following root treatment with our T. virens strain panel by using the cell expression by linear amplification and sequencing (CEL-Seq) method. Unlike the usual, full-length mRNA-sequencing, CEL-Seq library preparation, added with a unique primer designed with an anchored polyT for 3′ end tagging, retains only the 3′-most fragments of the mRNA transcripts in the sample, thereby allowing the sequencing of fewer reads to reach significance and providing strand specificity (74). cDNA libraries for sequencing were prepared using the CEL-Seq2 protocol (75) with several modifications. Instead of using single cells as input, 2 ng of purified RNA were taken as input for library preparation. Each initial RNA sample was barcoded with 3 different CEL-Seq primers, resulting in 3 technical repeats. For final library amplification, 10 cycles of PCR were performed. The CEL-Seq library was sequenced on an Illumina HiSeq 2500. The quality of the sequenced data was evaluated using FASTQC v.0.11.5. Per-base scores indicated high sequencing quality, with a small percentage of reads discarded due to adapter trimming, using the ‘trim galore’ tool.

### DEG analysis and visualization.

Mapping reads to the Solanum lycopersicum SL3.0 Ensembl reference genome was performed by Tophat2 v.2.1.0, with HTseq-count v. 0.11.2 used for gene counting and the DESeq2 R package v. 1.24.0 used for normalization and for differential expression analysis. In order to improve read counting, the annotation file was modified by the addition of 300 bp to well-annotated coding genes’ 3’UTR, except for where the elongation collided with the following gene or where there was an overlap between genes on the same strand.

All downstream analysis was based on DEG-to-treatment matrices with adjustments to analysis tools, using MSN-Excel or the web-based text and data tool package, http://www.molbiotools.com/. Volcano plots and principal component analysis plots were generated using basic plotting commands in R. Venn diagrams were calculated using the InteractiVenn (http://www.interactivenn.net/) online application (76) and were redesigned using the Lucidchart visual workspace (https://app.lucidchart.com/). Heat maps were generated using the Heatmapper.ca web server (77).

Functional analysis was performed by two complementary strategies: one, based on choosing specific genes and testing their expression patterns, and the other, based on performing gene ontology (GO) enrichment analysis. Gene lists that served for expression patterns analysis were compiled based on information mined from the scientific literature on plant defense and were compared to lists of predicted and manually curated genes from other transcriptomics studies (78–80) and databases, mainly the iTAK identifier and classifier (45). The final manually curated list includes gene annotations from the ‘corrected-to-batch effect’ DEG list (Table S4).

### Data availability.

The data that support the findings of this study are available in the supplemental material of this article.
