# Supplementary figures and images for: Gliotoxin, an Immunosuppressive Fungal Metabolite, Primes Plant Immunity: Evidence from Trichoderma virens-Tomato Interaction
Source: mBio. 2022 Jul 18;13(4):e00389-22. doi: 10.1128/mbio.00389-22 (PMC9426506; doi:10.1128/mbio.00389-22)

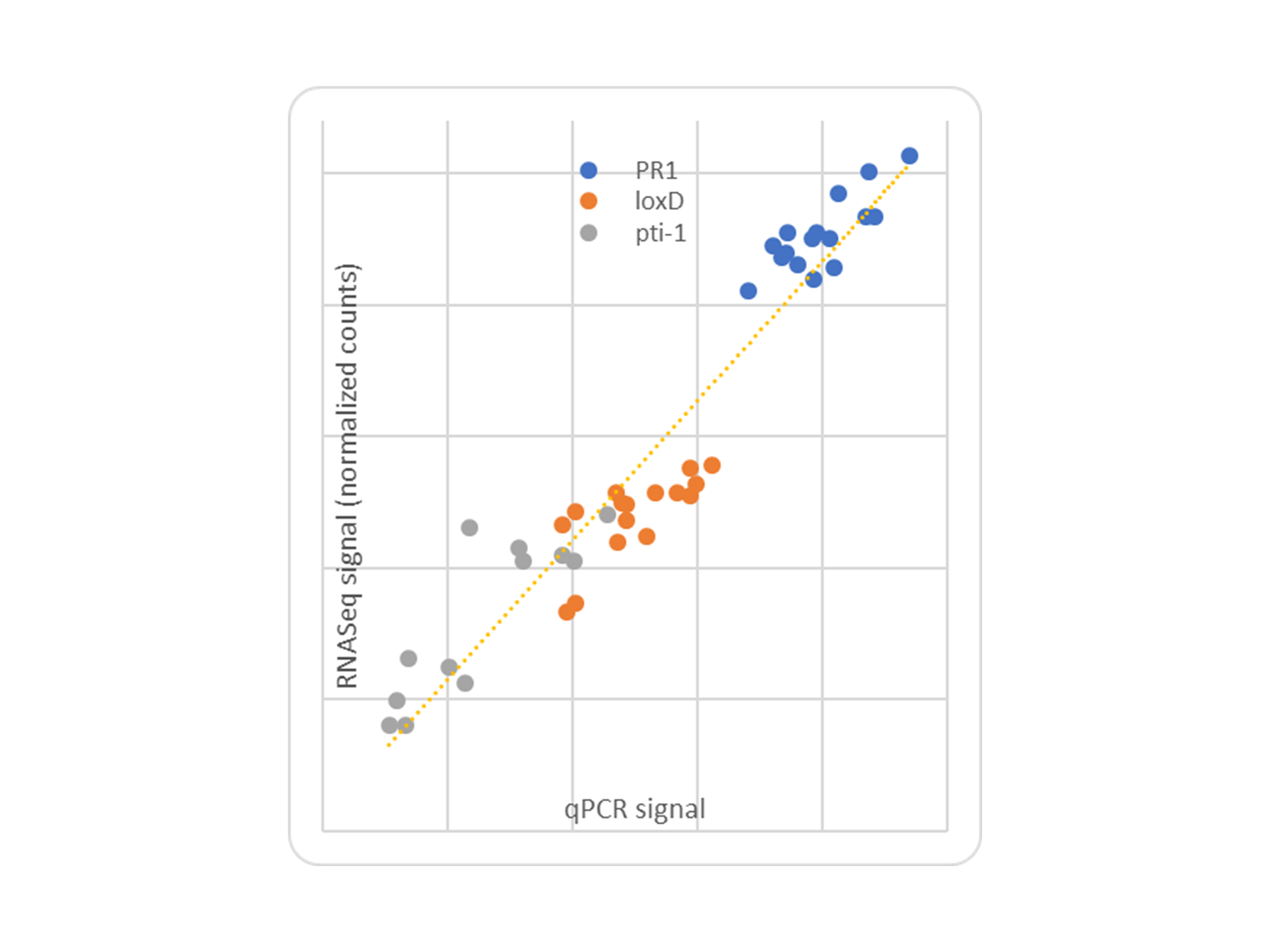

Supplement: FIG S1 [file mbio.00389-22-s0006.tif]

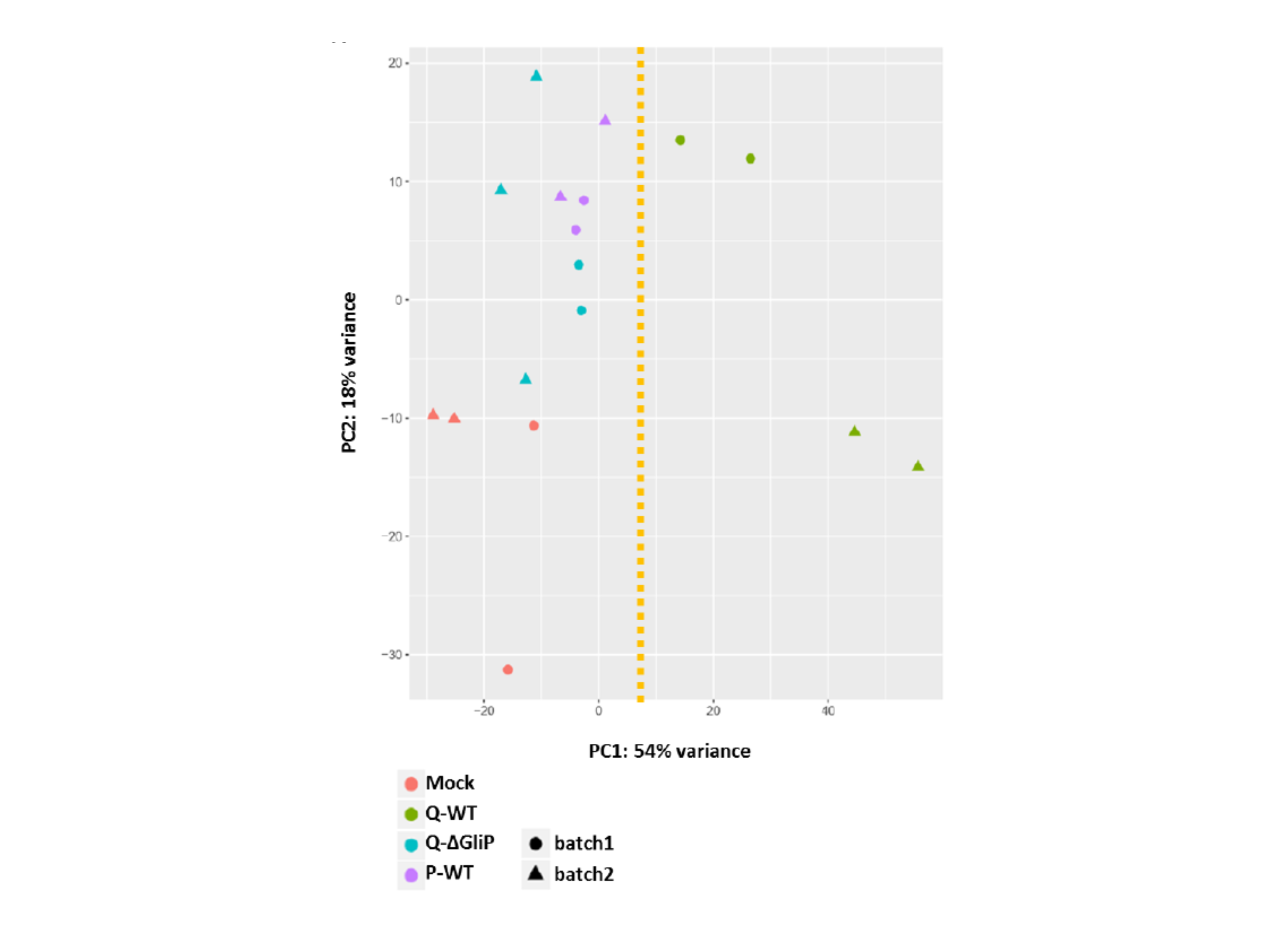

Supplement: FIG S2 [file mbio.00389-22-s0007.tif]

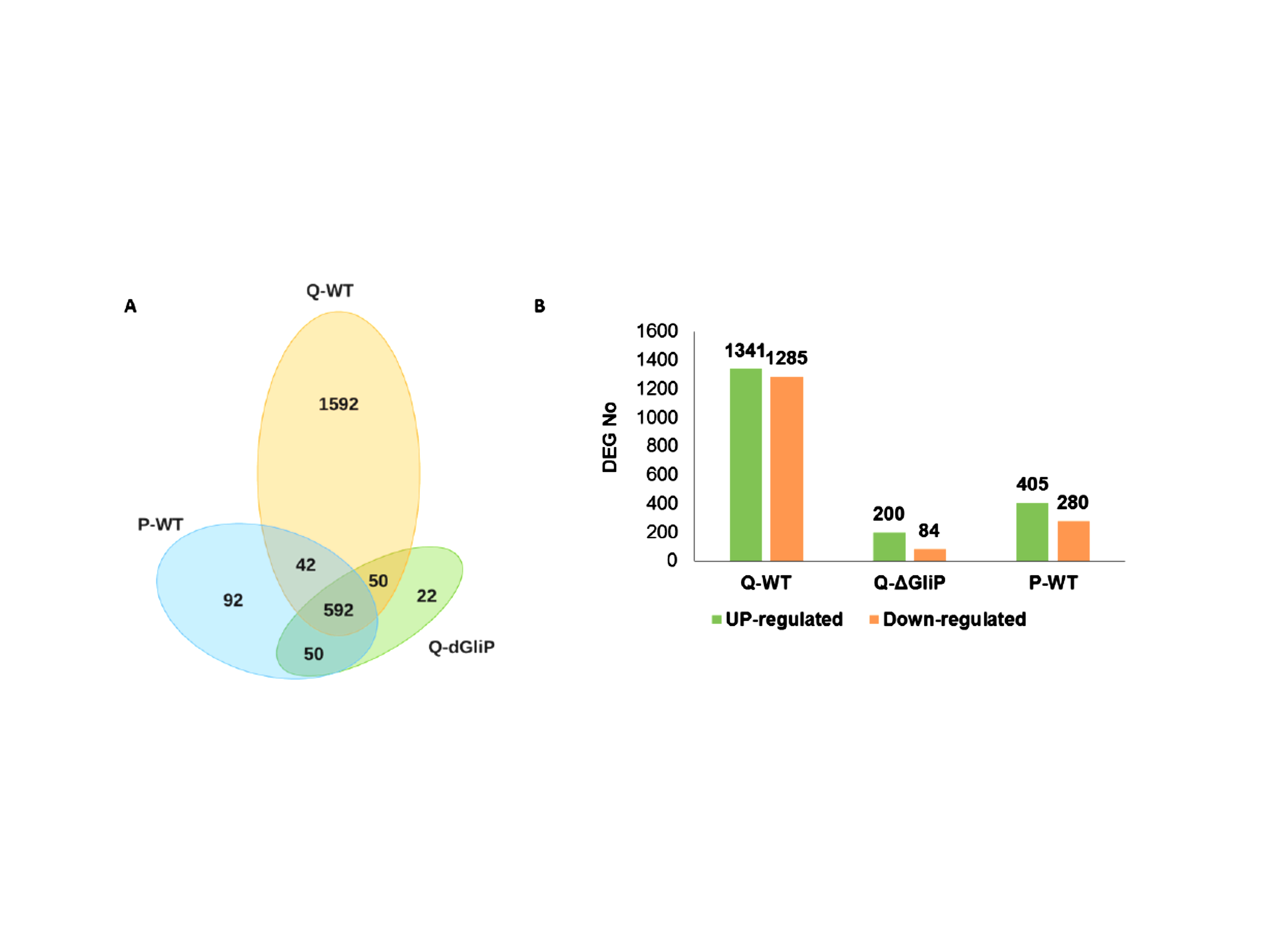

Supplement: FIG S3 [file mbio.00389-22-s0008.tif]

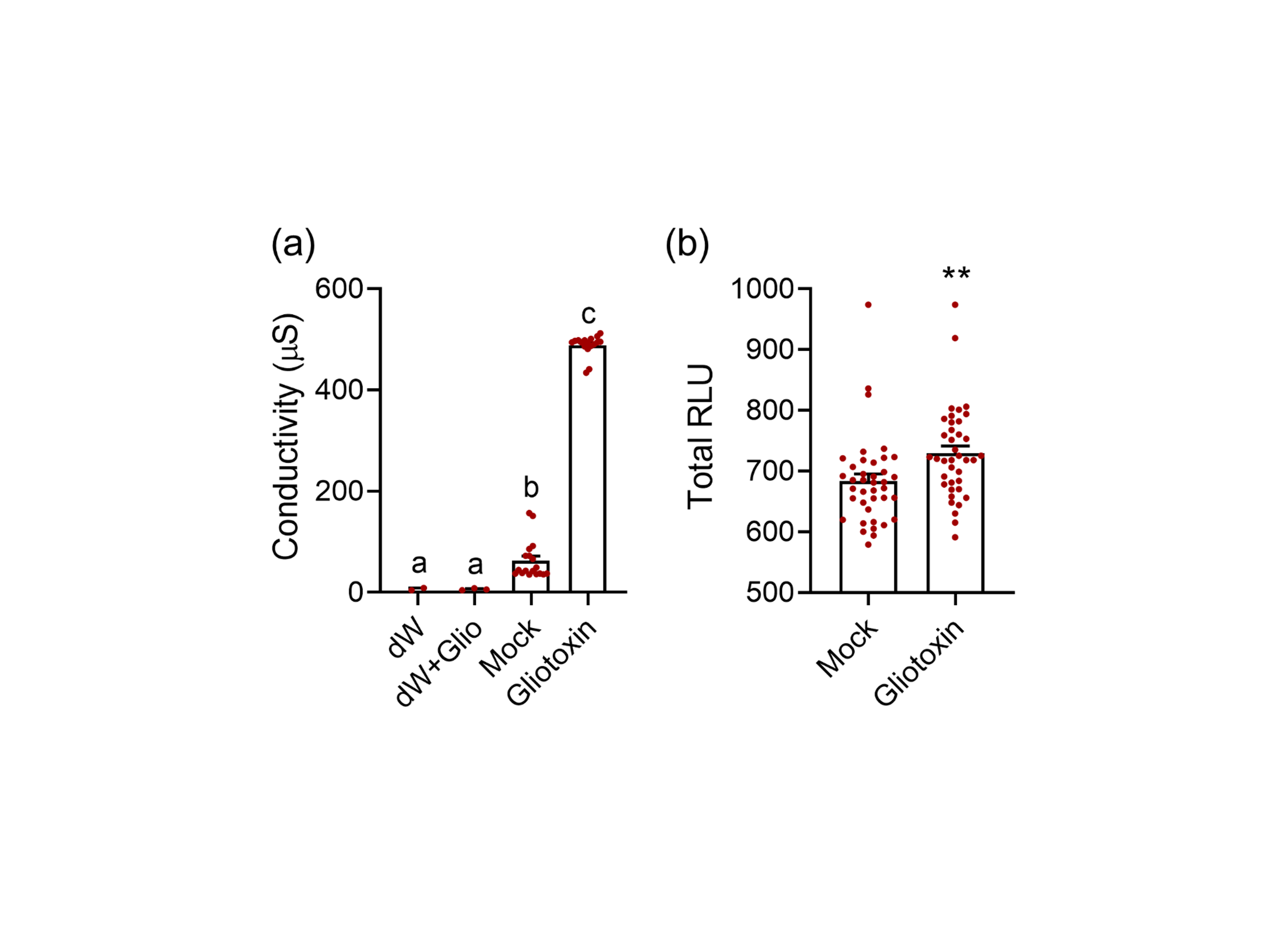

Supplement: FIG S4 [file mbio.00389-22-s0009.tif]
