# Supplementary material for: Gliotoxin, an Immunosuppressive Fungal Metabolite, Primes Plant Immunity: Evidence from Trichoderma virens-Tomato Interaction
Source: mBio. 2022 Jul 18;13(4):e00389-22. doi: 10.1128/mbio.00389-22 (PMC9426506; doi:10.1128/mbio.00389-22)
Supplement: TABLE S1 [file mbio.00389-22-s0001.docx]

**Table S1 –** Primers used for RT-qPCR (84)
