# Supplementary material for: Gliotoxin, an Immunosuppressive Fungal Metabolite, Primes Plant Immunity: Evidence from Trichoderma virens-Tomato Interaction
Source: mBio. 2022 Jul 18;13(4):e00389-22. doi: 10.1128/mbio.00389-22 (PMC9426506; doi:10.1128/mbio.00389-22)
Supplement: Supplementary file 6 [file mbio.00389-22-s0002.docx]

	Total # Genes	All Zero	Low Counts	Tested	Significant Up	Significant Down
Q-WT vs_mock	35, 039	11,266	7,695	16,079	2,310	2,571
Q-ΔGliP_vs_mock			11,720	12,054	502	855
P-WT_vs_mock			9,049	14,725	791	1,284
Q-ΔGliP _vs_ Q-WT			7,771	16,003	1,435	1,144
P-WT _vs_ Q-WT			7,841	15,933	971	676
P-WT _vs_ Q-ΔGliP			7,991	15,783	0	0
